# PROTOCOL: Guidance for stakeholder engagement in guideline development: A systematic review

**DOI:** 10.1002/cl2.1242

**Published:** 2022-05-11

**Authors:** Jennifer Petkovic, Alison Riddle, Lyubov Lytvyn, Joanne Khabsa, Elie A. Akl, Vivian Welch, Olivia Magwood, Pearl Atwere, Ian D. Graham, Sean Grant, Denny John, Srinivasa Vittal Katikireddi, Etienne Langlois, Reem A. Mustafa, Alex Todhunter‐Brown, Holger Schünemann, Maureen Smith, Airton T. Stein, Tom Concannon, Peter Tugwell

**Affiliations:** ^1^ Ottawa Centre for Health Equity Bruyère Research Institute Ottawa Canada; ^2^ School of Epidemiology and Public Health, Faculty of Medicine University of Ottawa Ottawa Canada; ^3^ Department of Clinical Epidemiology and Biostatistics McMaster University Hamilton Canada; ^4^ Clinical Research Institute American University of Beirut Medical Center Beirut Lebanon; ^5^ Department of Internal Medicine American University of Beirut Medical Center Beirut Lebanon; ^6^ Methods Centre Bruyère Research Institute Ottawa Canada; ^7^ C.T. Lamont Primary Health Care Research Centre Bruyère Research Institute Ottawa Canada; ^8^ Ottawa Centre for Health Equity Bruyère Research Institute Ottawa Canada; ^9^ Department of Social and Behavioral Sciences, Richard M. Fairbanks School of Public Health Indiana University Indianapolis Indiana USA; ^10^ Campbell Collaboration New Delhi India; ^11^ MRC/CSO Social and Public Health Sciences Unit University of Glasgow Glasgow UK; ^12^ Alliance for Health Policy and Systems Research World Health Organization Geneva Switzerland; ^13^ Nursing, Midwifery and Allied Health Professions Research Unit Glasgow Caledonian University Glasgow UK; ^14^ Departments of Health Research Methods Evidence, and Impact and of Medicine, McMaster University Hamilton Canada; ^15^ Canadian Organization for Rare Disorders Ottawa Canada; ^16^ Department of Public Health Universidade Federal de Ciências da Saúde Porto Alegre Brazil; ^17^ The RAND Corporation Boston Massachusetts USA; ^18^ Clinical Epidemiology Program Ottawa Hospital Research Institute Ottawa Canada

## Abstract

This is the protocol for a Campbell systematic review. The objectives are as follows: to identify, describe, and summarize existing guidance and methods for multistakeholder engagement throughout the health guideline development process.

## 1 BACKGROUND

### 1.1 The problem, condition or issue

Evidence‐informed healthcare guidelines evaluate and summarize the available evidence regarding patient care, public health and health systems, weigh the benefits and risks as well as acceptability, feasibility, and potential equity considerations that accompany all care and policy options and make recommendations (IOM, 2011). There is a moral imperative to include intended end users in the development of guidelines that may affect them. Stakeholder engagement, of all those potentially affected by the recommendations included in a guideline, is critical to ensuring the right issues are addressed and the right questions are asked (Gillard, 2012; Oliver, 2014). It may improve the relevancy, transparency and usefulness of guidelines (Esmail, 2015). Stakeholder engagement helps to ensure guideline acceptability and feasibility, support for its uptake and the practices recommended, and possible effects on adherence to any treatments and practices recommended (Carroll, 2017; Schunemann, 2014). Successful guideline development and implementation requires engagement of multiple stakeholders as well as shared solutions to improve health outcomes (Dunston, 2009; Kumarasame, 2016; Suman, 2016). Stakeholder groups potentially affected by guideline recommendations include patients, caregivers and the public, providers, payers, purchasers, product makers, policy makers, program managers, peer review editors, and principal investigators (Concannon, 2012; Tugwell, 2006). Clinicians and patients/consumers are the stakeholder groups predominantly engaged in guideline development and there has been little guidance developed for including other groups of stakeholders (Armstrong, 2017; Lavis, 2008; Oxman, 2006; van de Bovenkamp, 2015).

Effective stakeholder engagement should include the equitable inclusion of different groups and those who are known to be underrepresented. We use the term ‘underrepresented’ to refer to those individuals or groups who are (1) typically excluded from guideline development and implementation and (2) those who may experience inequities. These health inequities may exist for reasons such as a lack of inclusion in research, barriers to access of services, or because of other socially stratifying factors (Shi, 2014; Wallerstein, 2010). These factors can be described using the acronym PROGRESS‐Plus (O'Neill, 2014); Place of residence, Race/ethnicity/culture/language, Occupation, Gender/sex, Religion, Education, Socioeconomic status, Social capital, or other characteristic, such as age, in the guideline development and implementation process. Engagement of those affected by the guidelines and their recommendations ensures equity, diversity, and inclusion as well as social justice issues as it democratizes the guideline development process. It allows for stakeholder to participate in decision making for recommendations, programs, and policies that affect them.

There are many existing frameworks for how to develop guidelines, however, stakeholder engagement is often included as one step of the process and guidance for stakeholder engagement throughout the individual steps of the process may be lacking. Schunemann (2014) identified 18 topics in the guideline development process. Stakeholder engagement is one topic on the checklist (item 6). Selva et al. (2017) reviewed 56 guidance documents for guideline development and found that while 72% included the engagement of patients and their views in the guideline development process, few provided details on how it may be done. In light of the importance of stakeholder engagement in guideline development, this review aims to identify and describe existing guidance for stakeholder engagement that have been or that can be employed at different stages in the guideline development process. This includes identifying who to engage, when to engage them, and how to involve them throughout the process.

### 1.2 Definitions

For our purposes,

- Guidelines are ‘systematically developed evidence‐based statements which assist providers, recipients and other stakeholders informed decisions about appropriate health interventions’ (WHO, 2003).
- Stakeholders include patients, caregivers and the public, providers, payers of health services, payers of research, purchasers, product makers, policy makers, program managers, peer review editors, principal investigators or anyone who may be affected by a guideline.
- engagement entails an approach to ensure the contribution of stakeholders towards the development of the guideline, completion of any of the stages of the guideline, or dissemination of the guideline and its recommendations (Frank, 2020; Pollock, 2018). It is important to acknowledge that language in this field varies internationally and is continually evolving. Terms such as involvement, collaboration, or partnership have also been used to refer to engagement (Hoddinott, 2018). Within this review we use the term ‘engagement’.
- existing guidance describe systematic approaches for stakeholder engagement (Armstrong, 2017) and we will include descriptions of process(es), checklists, concepts, models, outlines, systems, plans and or overviews on engaging stakeholders in guideline development processes.

### 1.3 Description of the phenomena of interest

The role and value of stakeholder engagement in guideline development is increasing and therefore there is a need to identify and describe the existing guidance as this information will be useful for creating checklists for the meaningful engagement of multiple stakeholder groups in the guideline development process.

OIiver et al. have described that for committees, such as those involved in guideline development, multispeciality groups are recommended over singly speciality groups to allow for a greater range of opinion. While larger groups may be more difficult to manage and require a skilled and experienced facilitator, they also offer more opportunity for diversity among members and therefore may be more reliable, may enhance credibility and lead to widespread acceptance and implementation of decisions and recommendations (Oliver, 2018).

**Figure 1:**
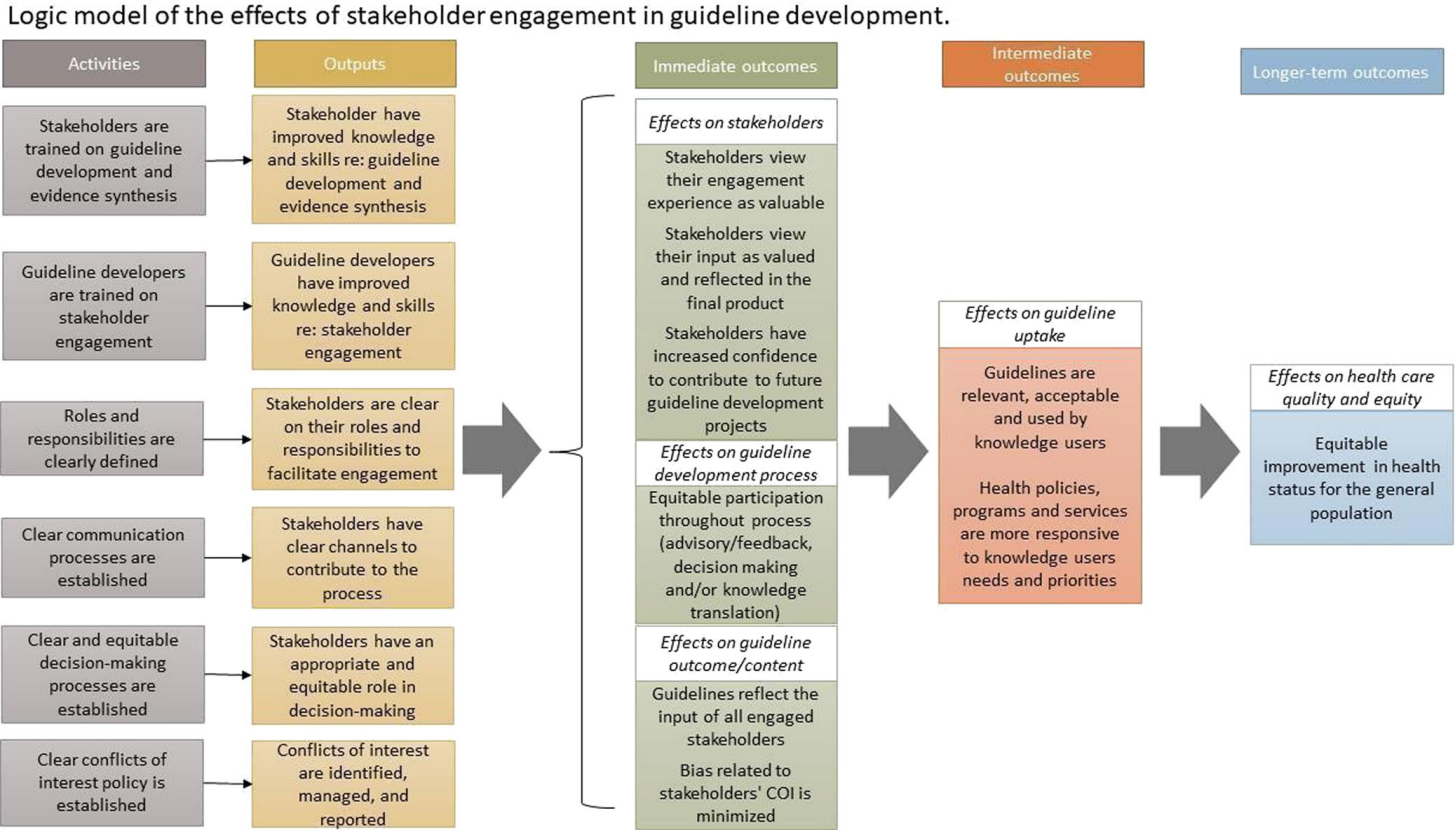
Logic model of the effects of stakeholder engagement in guideline development.

Stakeholder engagement may occur at various steps in the process. Stakeholder intensity can vary with some stakeholder groups being involved more intensely at certain points in the process (Crowe, 2017; Oliver, 2008; Pollock, 2019). The input of different stakeholder groups at different steps of the guideline process may have different affects on the development process. For example, including peer review editors early in the process ensures that the final guidance document is written according to standards required by the journals that may publish it, including patients during question formulation and the selection of outcomes ensures that their concerns are addresses, including program managers in steps related to evaluation and use ensures that the guideline and recommendations can be evaluated. In addition, they can be involved in advisory or feedback roles, where they receive information and are invited to provide their opinions and/or experience but for which there is no commitment to act on the feedback provided, or they may be involved in decision‐making in which their views influence the overall content of the guidelines. Engagement requires certain activities to be effective (Figure 1). Stakeholder engagement is a complex process and there are many factors that may influence stakeholders' ability to engage in the guideline development process as well as barriers to guideline developer's abilities to facilitate engagement. This scoping review focuses on existing guidance for how to effectively engage stakeholders in the process as described by the activities listed in our logic model (Figure 1) and mapped to the 18 topics of the GIN‐McMaster checklist.

### 1.4 Why it is important to do the review

Guidance for guideline development, including stakeholder engagement, exists. Many guideline development organizations provide guidance for guideline development but do not provide specific information about how and when to engage multiple stakeholders. The Guidelines International Network (GIN) has proposed key elements, including stakeholder engagement, that need to be considered in developing guidelines (Qaseem, 2012). In identifying components for guidelines on guideline development, Schunemann and colleagues (Schunemann, 2006) provide practical suggestions that World Health Organization (WHO) guideline working groups can build on in their work. The GRADE Evidence to Decision (EtD) frameworks suggests 10 criteria for those making different types of guideline decisions: priority of the problem, test accuracy, benefits and harms, certainty of the evidence, outcome importance, balance (between desirable and undesirable effects), resources use, equity, acceptability, feasibility (Alonso‐Coello, 2016). Similarly, the WHO‐Integrate evidence framework includes six criteria (balance of health benefits and harms, human rights and socio‐cultural acceptability, health equity, equality, and nondiscrimination, societal implications, financial and economic considerations, feasibility and health system considerations) and suggested methods to facilitate uptake of recommendations (Rehfuess, 2018). However, guidance specific to stakeholder engagement throughout each step of guideline development is lacking. Further, given the volume of existing guidance documents, it would be useful to synthesize all existing guidance about stakeholder engagement as well as highlight items that address equitable engagement.

Other reviews have assessed existing guidance for guideline development. For example, Schunemann et al. reviewed 35 guideline manuals (from 2003 to 2012) and developed a checklist of items to be considered in guideline development and implementation (Schunemann, 2014). This overview of guidance manuals is currently being updated. However, it does not specifically focus on stakeholder engagement throughout each step of the guideline development process.

In engaging stakeholders, many guideline developers focus on a single stakeholder, particularly patients, caregivers, or service users (e.g., persons without lived experience of the medical condition related to the guideline). For example, Armstrong and colleagues have developed a 10‐step model for engaging patients in guideline development. (Armstrong, 2017). We aim to additionally consider guidance for the interactions between multiple stakeholder groups on guideline development and implementation.

The GRADE (Grading of Recommendations Assessment, Development and Evaluation) system is internationally recognized as a standard for guideline development (Guyatt, 2008). The Multi‐Stakeholder Engagement (MuSE) working group is a global consortium established in 2015 to look into the development of methods for involving multiple stakeholders in health outcomes research (Concannon, 2019; Petkovic, 2020). One goal is to develop guidance for multistakeholder engagement in guideline development. To develop this guidance, the group will conduct this systematic review to identify existing guidance for stakeholder engagement in guideline development in parallel with three other reviews focused on (a) barriers and facilitators to stake holder engagement, (b) disclosure, management and reporting of potential conflicts of interest during guideline development and c) the impact of stakeholder engagement on the guideline development process. Currently, the GRADE Handbook states that ‘the guideline panel and supporting groups … work collaboratively, informed through consumer and stakeholder involvement’ (Schunemann, 2013) but does not provide specific guidance on how this should be achieved. The results of this review, together with the other three in the series, will be used to inform the development of guidance for multistakeholder engagement in guideline development and implementation. This guidance will be official GRADE Working Group guidance.

This review will answer the questions of what guidance exists for stakeholder engagement in health guideline development and for which steps of the guideline process. The findings of this systematic review, will be used to identify and summarize existing guidance for engaging with different stakeholders in the various stages of the guideline development process. Together with the results of the other reviews in this series, the results and the GIN‐McMaster extension checklist, may be used to assist organizations who develop healthcare, public health, and health policy guidelines, such as the World Health Organization, to involve multiple stakeholders in the guideline development process to ensure the development of relevant, high quality, and transparent guidelines. This protocol and the others in this series have been developed with input from all members of the MuSE Consortium as well as stakeholders representing our identified stakeholder groups.

## 2 OBJECTIVES

The objective of this review is to identify, describe, and summarize existing guidance and methods for multistakeholder engagement throughout the health guideline development process.

## 3 METHODS

### 3.1 Criteria for including and excluding studies

#### 3.1.1 Types of study designs

Our methods will follow the Cochrane Handbook for Systematic Reviews of Interventions and the Handbook for Qualitative Research, as appropriate (Higgins, 2019; Sandelowski, 2007).

To be included, papers must describe the process or methods for stakeholder engagement in guideline development using the definition of guideline described above. We will include quantitative, qualitative, and mixed‐method studies. Eligible study designs include: randomized controlled trials (RCTs), quasi‐randomized controlled trials, and nonrandomized studies (e.g., before and after studies, cohort studies, cross‐sectional studies) as well as theoretical papers, process evaluation studies, and qualitative studies. Mixed methods studies that apply a combination of the eligible quantitative and qualitative study designs and report on qualitative and quantitative outcomes separately will be eligible.

We will exclude editorials, commentaries, protocols, and conference abstracts. We will also exclude guidance provided in handbooks and other publications produced by organizations typically involved in health guideline development, such as the World Health Organization (WHO), National Health and Medical Research Council (NHMRC, Australia), National Institute for Health and Care Excellence (NICE, UK). Stakeholder engagement in the social care context will be excluded.

#### 3.1.2 Population of interest

We have identified 13 types of stakeholders whose input can enhance the relevance and uptake of research (Concannon, 2012, 2019; Tugwell, 2006). For the purposes of this review, have grouped them as follows:

- Patients, caregivers, and patient advocates
- the Public
- Providers of health care
- Payers of health services
- Payers of research
- Policymakers
- Program managers
- Product makers
- Purchasers
- Principal investigators and their research teams, and
- Peer review editors

Terms such as involvement, collaboration, or partnership (Hoddinott, 2018) have also been used in reference to engagement. Herein, we will use the term ‘stakeholder engagement’.

As an example, a potentially included study describes the development of a 10‐step framework for patient engagement in clinical practice guideline development (Armstrong, 2017).

#### 3.1.3 Phenomena of interest

We will focus on studies that describe stakeholder engagement in the guideline development process. We will include studies describing the development and/or implementation of a process for stakeholder engagement in guideline development. Guidelines on healthcare‐related issues, and clinical, public, or social health will be considered.

We will include papers discussing any topic of the guideline development process as described by the GIN‐McMaster Guideline Development Checklist (Schunemann, 2014):

1. Organization, budget, planning and training
2. Priority‐setting
3. Guideline group membership
4. Establishing guideline group processes
5. Identifying target audience and topic selection
6. Consumer and stakeholder involvement
7. Conflict of interest considerations
8. Question generation
9. Considering importance of outcomes and interventions, values, preferences, and utilities
10. Deciding what evidence to include and searching for evidence
11. Summarizing evidence and considering additional information
12. Judging quality, strength or certainty of body of evidence
13. Developing recommendations and determining their strength
14. Wording of recommendations and of considerations about implementation, feasibility and equity
15. Reporting and peer review
16. Dissemination and implementation
17. Evaluation and use
18. Updating

#### 3.1.4 Types of outcome measures

Outcomes for this review include

- Methods for identifying stakeholders at each step of the guideline development process
- Methods for engaging stakeholders at each step of the guideline development process
- Methods for training stakeholder to facilitate participation
- Frequency and level of engagement of stakeholders at each step of the guideline development process
- Level of engagement (Crowe, 2017; Oliver, 2008; Pollock, 2019) in each step of the guideline development process (advisory/feedback, decision‐making)
- How stakeholders contributed at each step of the guideline development process
- Methods for resolving disagreements
- Evaluation of engagement processes

#### 3.1.5 Types of settings

We will place no restrictions on setting.

### 3.2 Search strategy

We will develop one comprehensive search strategy for all four systematic reviews in consultation with a medical librarian which will be reviewed by a second medical librarian. We will search the following databases: MEDLINE (OVID), CINAHL (EBSCO), EMBASE (OVID), PsycInfo (OVID) and SCOPUS. Limits will not be placed on date, study design or language.

We will conduct an extensive grey literature search. We will search the websites of agencies who actively engage stakeholder groups in their work such as the Agency for Healthcare Research and Quality (AHRQ), CIHR Strategy for Patient‐Oriented Research (SPOR), INVOLVE, Guidelines Internation Network (G‐I‐N), the National Institute for Health and Care Excellence, and the Patient‐Centred Outcomes Research Institute (PCORI). We will also search the websites of guideline‐producing agencies, such as the American Academy of Paediatrics, Australia's National Health Medical Research Council (NHMRC), and the World Health Organization (WHO) including Latin American and Caribbean Health Sciences Literature (LILACS).

We will solicit suggestions for additional grey literature sources from the MuSE working group members and via social media, such as Twitter.

### 3.3 Description of methods used in primary research

It is expected that included papers will describe frameworks or guidance for who, when and how to engage stakeholders in guideline development and implementation.

### 3.4 Details of study coding categories

Two reviewers will independently screen titles and abstracts to identify relevant studies meeting the pre‐specified inclusion criteria. The full text of potentially included studies will be screened independently by two authors. We will use Covidence software (https://www.covidence.org/) for screening of studies. All completed studies will be included if they meet the inclusion criteria listed above.

The data extraction form will be pre‐tested and extracted independently in duplicate by two reviewers using Eppi‐Reviewer to facilitate qualitative synthesis (described below) (Thomas, 2010).

Disagreements will be resolved by discussion and with a third member of the research team when necessary.

We will extract details related to guidance for stakeholder engagement for each of our identified stakeholder groups for each of the 18 topics (146 steps) of the GIN‐McMaster checklist (Table 1). We will also extract data on:

- General study characteristics such as study design and year of publication
- Stakeholder groups and definition of stakeholder
- Definition of engagement
- Methods for identifying stakeholders
- Characteristics of stakeholder panel
- Whether stakeholders were part of the guideline development panel or external
- Methods for recruitment
- Methods for balancing stakeholder group representation
- Methods for engaging/method of communication
- Frequency of engagement
- Level of engagement (advisory/feedback or decision‐making)
- How stakeholders contributed
- Methods for resolving disagreement

**Table 1:** Framework for data extraction.

	Stakeholder groups									
Guideline development topics	Patients, caregivers, patient organizations	Public	Providers	Principal investigators + team	Policy makers	Program managers	Payers/Purchasers of health services	Payers of health research	Peer Review editors	Product makers
1. Organization, budget, planning and training										
2. Priority setting										
3. Guideline group membership										
4. Establishing guideline group processes										
5. Identifying target audience and topic selection										
6. Consumer and stakeholder involvement										
7. Conflict of interest (COI) considerations										
8. (PICO) question generation										
9. Considering importance of outcomes and interventions, values, preferences, and utilities										
10. Deciding what evidence to include and searching for evidence										
11. Summarizing evidence and considering additional information										
12. Judging quality, strength or certainty of a body of evidence										
13. Developing recommendations and determining their strength										
14. Wording of recommendations and of considerations of implementation, feasibility and equity										
15. Reporting and peer review										
16. Dissemination and implementation										
17. Evaluation and use										
18. Updating										

### 3.5 Quality assessment

We will assess the quality of our included papers using the criteria: practicality, relevance, and legitimacy as described in the tool developed by Movsisyan et al. (2019). This tool assesses, for example, whether the paper in describes key concepts clearly, whether the guidance can be easily applied without additional information, whether there is information related to its adaptation to other settings, and whether the underlying theory and principles have been described (see Table 2). We will also assess whether the development of the guidance has included input from relevant stakeholders.

**Table 2:** Quality appraisal criteria.

Quality Appraisal (adapted from Movsisyan, 2019)	Rating	Comments
*Practicality*		
Key concepts/nomenclature and their definitions related to guidance development	−/+/++	
Definition and description—understandability and clarity of key constructs		
Guiding question:		
Are the key items/guidance clearly specified?		
Ease of use and operationalisability	−/+/++	
Guiding questions:		
Can the guidance be applied easily without the need to search for additional information?		
Can the guidance be adequately operationalised?		
Comprehensiveness	−/+/++	
Guiding questions:		
Does the guidance thoroughly describe information related to adaptation in a new setting?		
Does the guidance thoroughly describe information related to evaluation in a new setting?		
*Relevance*		
Relevance for use in different settings and with different stakeholders	−/+/++	
Guiding question:		
Can the guidance be applied to different types of guidelines?		
Can the guidance be used in different settings?		
Can the guidance be adapted to additional stakeholders?		
*Legitimacy*		
Legitimacy—Scientific basis and development process	−/+/++	
Guiding questions:		
Does the guidance describe its underlying theory and principles?		
Does the guidance describe a rigorous development process (such as a comprehensive literature review and/or a consensus‐based methodology)		

Abbreviations: −, not addressed; +, partially addressed; ++, fully addressed.

Quality will be assessed independently, in duplicate, by two authors and any discrepancies will be resolved by consensus and consultation with a third author, when necessary.

### 3.6 Statistical procedures and conventions

We will not have data for statistical analysis.

### 3.7 Treatment of qualitative research

We will narratively summarize the existing guidance identified in our included studies by mapping the descriptions of stakeholder engagement in the included papers to the 18 topics (146 steps) of the guideline development process outlined by the GIN‐McMaster checklist (Schunemann, 2014). We will present this information as a matrix indicating the guidance that exists for each stakeholder group and for each step of guideline development.

We will use thematic synthesis to combine guidance for stakeholder involvement in each step.

We will follow the guidance for qualitative synthesis in systematic reviews as outlined by Thomas (2008) which includes coding the text and developing descriptive themes (Thomas, 2008). This involves translating concepts from one study to another and looking for similarities and differences between codes to allow for grouping concepts into themes for analysis (Thomas, 2008).

## SOURCES OF SUPPORT

Internal sources

- No sources of support provided

External sources

- Canadian Institutes of Health Research, Canada

CIHR Project Grant

## CONTRIBUTIONS OF AUTHORS

Conceiving the review: PT, VW, TC, JP

Designing the review: JP, AR, VW, PT, TC

Coordinating the review: JP, AR, PA

Writing the protocol: JP, PA, AR, JK, LL

Providing general advice on the review: JK, LL, EA, VW, OM, IDG, SG, DJ, SVK, AS, HS, TC, PT

Securing funding for the review: PT, VW, JP

## DECLARATIONS OF INTEREST

SG's spouse is a salaried employee of Eli Lilly and Company and owns stock. SG has accompanied his spouse on company‐sponsored travel. SVK acknowledges research funding from the UK Medical Research Council, Scottish Government Chief Scientist Office and a NRS Senior Clinical Fellowship. SVK is an honorary Consultant in Public Health at Public Health Scotland and has provided unpaid advice to Obesity Action Scotland, Scottish Government and UK Government. VW is editor in chief of the Campbell Collaboration. This review will be handled by an independent editor, and the cochairs of the relevant group will act in lieu of editor in chief. TC has developed and published several peer‐reviewed publications that could potentially be included in the review. TC currently holds one research contract with the Patient‐Centred Outcomes Research Institute and another with the Pharmaceutical Research and Manufacturers of America Foundation that address a similar topic.

## Supporting information

Supporting information.
