# Corrigendum

**DOI:** 10.1002/cl2.1307

**Published:** 2023-02-05

1

**Corrigendum to “PROTOCOL: Guidance for stakeholder engagement in guideline development: A systematic review”**

The title of the above Protocol published with Campbell Systematic Reviews should correctly read as:

**PROTOCOL: Guidance for stakeholder engagement in guideline development: A scoping review**

The original version is also corrected and can be found on the below link: https://doi.org/10.1002/cl2.1242. The authors apologize for this error.
