# Supplementary material for: PROTOCOL: Guidance for stakeholder engagement in guideline development: A systematic review
Source: Campbell Syst Rev. 2022 May 11;18(2):e1242. doi: 10.1002/cl2.1242 (PMC9096120; doi:10.1002/cl2.1242)
Supplement: Supplementary file 1 — Supporting information. [file CL2-18-e1242-s001.docx]

# Appendices

## 1 MEDLINE Search Strategy

1 Stakeholder Participation/

2 patient participation/

3 consumer participation/

4 Community-Based Participatory Research/

5 (coproduction or co-production).ti,ab,kf.

6 ((stakeholder* or advisor* or reference* or expert* or consultation* or steering) adj2 (group* or panel*)).ti,ab,kf.

7 ((stakeholder* or patient* or consumer* or public or caregiver* or care-giver* or communit* or citizen* or user* or service-user* or end-user* or clinician* or doctor* or physician* or nurse* or policymaker* or policy-maker* or funder* or indust* or pharmaceutical) adj2 (engag* or involv* or input or participat* or collaborat*)).ti,ab,kf.

8 or/1-7

9 practice guidelines as topic/

10 Guideline*.ti.

11 Guidance.ti,kf.

12 Clinical guideline*.ti,ab,kf.

13 Clinical Practice Guideline*.ti,ab,kf.

14 or/9-13

15 8 and 14

## 2 CINAHL Search Strategy

| **#** | **Query** | **Limiters/Expanders** | **Last Run Via** |
| --- | --- | --- | --- |
| S11 | S5 AND S10 | Search modes - Boolean/Phrase | Interface - EBSCOhost Research Databases Search Screen - Advanced Search Database - CINAHL Complete |
| S10 | S6 OR S7 OR S8 OR S9 | Search modes - Boolean/Phrase | Interface - EBSCOhost Research Databases Search Screen - Advanced Search Database - CINAHL Complete |
| S9 | (clinical OR practice) N1 guideline* | Search modes - Boolean/Phrase | Interface - EBSCOhost Research Databases Search Screen - Advanced Search Database - CINAHL Complete |
| S8 | TI Guidance OR SU Guidance | Search modes - Boolean/Phrase | Interface - EBSCOhost Research Databases Search Screen - Advanced Search Database - CINAHL Complete |
| S7 | TI Guideline* | Search modes - Boolean/Phrase | Interface - EBSCOhost Research Databases Search Screen - Advanced Search Database - CINAHL Complete |
| S6 | MH Practice Guidelines | Search modes - Boolean/Phrase | Interface - EBSCOhost Research Databases Search Screen - Advanced Search Database - CINAHL Complete |
| S5 | S1 OR S2 OR S3 OR S4 | Search modes - Boolean/Phrase | Interface - EBSCOhost Research Databases Search Screen - Advanced Search Database - CINAHL Complete |
| S4 | (stakeholder* OR patient* OR consumer* OR public OR caregiver* OR communit* OR citizen* OR user* OR service-user* OR end-user* OR clinician* OR doctor* OR physician* OR nurse* OR policymaker* OR funder* OR industry OR pharmaceutical) N2 (engag* OR involv* OR input OR participat*) | Search modes - Boolean/Phrase | Interface - EBSCOhost Research Databases Search Screen - Advanced Search Database - CINAHL Complete |
| S3 | (stakeholder* OR advisor* OR reference* OR expert* OR consultation OR steering) N2 (group* OR panel) | Search modes - Boolean/Phrase | Interface - EBSCOhost Research Databases Search Screen - Advanced Search Database - CINAHL Complete |
| S2 | coproduction OR co-production | Search modes - Boolean/Phrase | Interface - EBSCOhost Research Databases Search Screen - Advanced Search Database - CINAHL Complete |
| S1 | MH Consumer Participation | Search modes - Boolean/Phrase | Interface - EBSCOhost Research Databases Search Screen - Advanced Search Database - CINAHL Complete |

## 3 EMBASE Search Strategy

1. stakeholder engagement/

2. patient participation/

3. consumer/

4. participatory research/

5. (coproduction or co-production).ti,ab,kw.

6. ((stakeholder* or advisor* or reference* or expert* or consultation* or steering) adj2 (group* or panel*)).ti,ab,kw.

7. ((stakeholder* or patient* or consumer* or public or caregiver* or care-giver* or communit* or citizen* or user* or service-user* or end-user* or clinician* or doctor* or physician* or nurse* or policymaker* or policy-maker* or funder* or indust* or pharmaceutical) adj2 (engag* or involv* or input or participat* or collaborat*)).ti,ab,kw.

8.  or/1-7

9.   practice guideline/

10.  Guideline*.ti.

11.  Guidance.ti,kw.

12.  Clinical guideline*.ti,ab,kw.

13.  Clinical Practice Guideline*.ti,ab,kw.

14.  or/9-13

15.   8 and 14

## 4 PsycINFO Search Strategy

1 Client Participation/

2 Stakeholder/ and Involvement/

3 Stakeholder/ and Participation/

4 coproduction.ti,ab.

5 co-production.ti,ab.

6 (stakeholder* adj2 group*).mp. [mp=title, abstract, heading word, table of contents, key concepts, original title, tests & measures]

7 (stakeholder* adj2 panel).mp. [mp=title, abstract, heading word, table of contents, key concepts, original title, tests & measures]

8 (advisor* adj2 group*).mp. [mp=title, abstract, heading word, table of contents, key concepts, original title, tests & measures]

9 (advisor* adj2 panel).mp. [mp=title, abstract, heading word, table of contents, key concepts, original title, tests & measures]

10 (reference* adj2 group*).mp. [mp=title, abstract, heading word, table of contents, key concepts, original title, tests & measures]

11 (reference* adj2 panel).mp. [mp=title, abstract, heading word, table of contents, key concepts, original title, tests & measures]

12 (expert* adj2 group*).mp. [mp=title, abstract, heading word, table of contents, key concepts, original title, tests & measures]

13 (expert* adj2 panel).mp. [mp=title, abstract, heading word, table of contents, key concepts, original title, tests & measures]

14 (consultation adj2 group*).mp. [mp=title, abstract, heading word, table of contents, key concepts, original title, tests & measures]

15 (consultation adj2 panel).mp. [mp=title, abstract, heading word, table of contents, key concepts, original title, tests & measures]

16 (steering adj2 group*).mp. [mp=title, abstract, heading word, table of contents, key concepts, original title, tests & measures]

17 (steering adj2 panel).mp. [mp=title, abstract, heading word, table of contents, key concepts, original title, tests & measures]

18 (stakeholder* adj2 engag*).mp. [mp=title, abstract, heading word, table of contents, key concepts, original title, tests & measures]

19 (stakeholder* adj2 involv*).mp. [mp=title, abstract, heading word, table of contents, key concepts, original title, tests & measures]

20 (stakeholder* adj2 input).mp. [mp=title, abstract, heading word, table of contents, key concepts, original title, tests & measures]

21 (stakeholder* adj2 participat*).mp. [mp=title, abstract, heading word, table of contents, key concepts, original title, tests & measures]

22 (patient* adj2 engag*).mp. [mp=title, abstract, heading word, table of contents, key concepts, original title, tests & measures]

23 (patient* adj2 involv*).mp. [mp=title, abstract, heading word, table of contents, key concepts, original title, tests & measures]

24 (patient* adj2 input).mp. [mp=title, abstract, heading word, table of contents, key concepts, original title, tests & measures]

25 (patient* adj2 participat*).mp. [mp=title, abstract, heading word, table of contents, key concepts, original title, tests & measures]

26 (consumer* adj2 engag*).mp. [mp=title, abstract, heading word, table of contents, key concepts, original title, tests & measures]

27 (consumer* adj2 involv*).mp. [mp=title, abstract, heading word, table of contents, key concepts, original title, tests & measures]

28 (consumer* adj2 input).mp. [mp=title, abstract, heading word, table of contents, key concepts, original title, tests & measures]

29 (consumer* adj2 participat*).mp. [mp=title, abstract, heading word, table of contents, key concepts, original title, tests & measures]

30 (public adj2 engag*).mp. [mp=title, abstract, heading word, table of contents, key concepts, original title, tests & measures]

31 (public adj2 involv*).mp. [mp=title, abstract, heading word, table of contents, key concepts, original title, tests & measures]

32 (public adj2 input).mp. [mp=title, abstract, heading word, table of contents, key concepts, original title, tests & measures]

33 (public adj2 participat*).mp. [mp=title, abstract, heading word, table of contents, key concepts, original title, tests & measures]

34 (caregiver* adj2 engag*).mp. [mp=title, abstract, heading word, table of contents, key concepts, original title, tests & measures]

35 (caregiver* adj2 involv*).mp. [mp=title, abstract, heading word, table of contents, key concepts, original title, tests & measures]

36 (caregiver* adj2 input).mp. [mp=title, abstract, heading word, table of contents, key concepts, original title, tests & measures]

37 (caregiver* adj2 participat*).mp. [mp=title, abstract, heading word, table of contents, key concepts, original title, tests & measures]

38 (communit* adj2 engag*).mp. [mp=title, abstract, heading word, table of contents, key concepts, original title, tests & measures]

39 (communit* adj2 involv*).mp. [mp=title, abstract, heading word, table of contents, key concepts, original title, tests & measures]

40 (communit* adj2 input).mp. [mp=title, abstract, heading word, table of contents, key concepts, original title, tests & measures]

41 (communit* adj2 participat*).mp. [mp=title, abstract, heading word, table of contents, key concepts, original title, tests & measures]

42 (citizen* adj2 engag*).mp. [mp=title, abstract, heading word, table of contents, key concepts, original title, tests & measures]

43 (citizen* adj2 involv*).mp. [mp=title, abstract, heading word, table of contents, key concepts, original title, tests & measures]

44 (citizen* adj2 input).mp. [mp=title, abstract, heading word, table of contents, key concepts, original title, tests & measures]

45 (citizen* adj2 participat*).mp. [mp=title, abstract, heading word, table of contents, key concepts, original title, tests & measures]

46 (user* adj2 engag*).mp. [mp=title, abstract, heading word, table of contents, key concepts, original title, tests & measures]

47 (user* adj2 involv*).mp. [mp=title, abstract, heading word, table of contents, key concepts, original title, tests & measures]

48 (user* adj2 input).mp. [mp=title, abstract, heading word, table of contents, key concepts, original title, tests & measures]

49 (user* adj2 participat*).mp. [mp=title, abstract, heading word, table of contents, key concepts, original title, tests & measures]

50 (service-user* adj2 engag*).mp. [mp=title, abstract, heading word, table of contents, key concepts, original title, tests & measures]

51 (service-user* adj2 involv*).mp. [mp=title, abstract, heading word, table of contents, key concepts, original title, tests & measures]

52 (service-user* adj2 input).mp. [mp=title, abstract, heading word, table of contents, key concepts, original title, tests & measures]

53 (service-user* adj2 participat*).mp. [mp=title, abstract, heading word, table of contents, key concepts, original title, tests & measures]

54 (end-user* adj2 engag*).mp. [mp=title, abstract, heading word, table of contents, key concepts, original title, tests & measures]

55 (end-user* adj2 involv*).mp. [mp=title, abstract, heading word, table of contents, key concepts, original title, tests & measures]

56 (end-user* adj2 input).mp. [mp=title, abstract, heading word, table of contents, key concepts, original title, tests & measures]

57 (end-user* adj2 participat*).mp. [mp=title, abstract, heading word, table of contents, key concepts, original title, tests & measures]

58 (clinician* adj2 engag*).mp. [mp=title, abstract, heading word, table of contents, key concepts, original title, tests & measures]

59 (clinician* adj2 involv*).mp. [mp=title, abstract, heading word, table of contents, key concepts, original title, tests & measures]

60 (clinician* adj2 input).mp. [mp=title, abstract, heading word, table of contents, key concepts, original title, tests & measures]

61 (clinician* adj2 participat*).mp. [mp=title, abstract, heading word, table of contents, key concepts, original title, tests & measures]

62 (doctor* adj2 engag*).mp. [mp=title, abstract, heading word, table of contents, key concepts, original title, tests & measures]

63 (doctor* adj2 involv*).mp. [mp=title, abstract, heading word, table of contents, key concepts, original title, tests & measures]

64 (doctor* adj2 input).mp. [mp=title, abstract, heading word, table of contents, key concepts, original title, tests & measures]

65 (doctor* adj2 participat*).mp. [mp=title, abstract, heading word, table of contents, key concepts, original title, tests & measures]

66 (physician* adj2 engag*).mp. [mp=title, abstract, heading word, table of contents, key concepts, original title, tests & measures]

67 (physician* adj2 involv*).mp. [mp=title, abstract, heading word, table of contents, key concepts, original title, tests & measures]

68 (physician* adj2 input).mp. [mp=title, abstract, heading word, table of contents, key concepts, original title, tests & measures]

69 (physician* adj2 participat*).mp. [mp=title, abstract, heading word, table of contents, key concepts, original title, tests & measures]

70 (nurse* adj2 engag*).mp. [mp=title, abstract, heading word, table of contents, key concepts, original title, tests & measures]

71 (nurse* adj2 involv*).mp. [mp=title, abstract, heading word, table of contents, key concepts, original title, tests & measures]

72 (nurse* adj2 input).mp. [mp=title, abstract, heading word, table of contents, key concepts, original title, tests & measures]

73 (nurse* adj2 participat*).mp. [mp=title, abstract, heading word, table of contents, key concepts, original title, tests & measures]

74 (policymaker* adj2 engag*).mp. [mp=title, abstract, heading word, table of contents, key concepts, original title, tests & measures]

75 (policymaker* adj2 involv*).mp. [mp=title, abstract, heading word, table of contents, key concepts, original title, tests & measures]

76 (policymaker* adj2 input).mp. [mp=title, abstract, heading word, table of contents, key concepts, original title, tests & measures]

77 (policymaker* adj2 participat*).mp. [mp=title, abstract, heading word, table of contents, key concepts, original title, tests & measures]

78 (funder* adj2 engag*).mp. [mp=title, abstract, heading word, table of contents, key concepts, original title, tests & measures]

79 (funder* adj2 involv*).mp. [mp=title, abstract, heading word, table of contents, key concepts, original title, tests & measures]

80 (funder* adj2 input).mp. [mp=title, abstract, heading word, table of contents, key concepts, original title, tests & measures]

81 (funder* adj2 participat*).mp. [mp=title, abstract, heading word, table of contents, key concepts, original title, tests & measures]

82 (industry adj2 engag*).mp. [mp=title, abstract, heading word, table of contents, key concepts, original title, tests & measures]

83 (industry adj2 involv*).mp. [mp=title, abstract, heading word, table of contents, key concepts, original title, tests & measures]

84 (industry adj2 input).mp. [mp=title, abstract, heading word, table of contents, key concepts, original title, tests & measures]

85 (industry adj2 participat*).mp. [mp=title, abstract, heading word, table of contents, key concepts, original title, tests & measures]

86 (pharmaceutical adj2 engag*).mp. [mp=title, abstract, heading word, table of contents, key concepts, original title, tests & measures]

87 (pharmaceutical adj2 involv*).mp. [mp=title, abstract, heading word, table of contents, key concepts, original title, tests & measures]

88 (pharmaceutical adj2 input).mp. [mp=title, abstract, heading word, table of contents, key concepts, original title, tests & measures]

89 (pharmaceutical adj2 participat*).mp. [mp=title, abstract, heading word, table of contents, key concepts, original title, tests & measures]

90 1 or 2 or 3 or 4 or 5 or 6 or 7 or 8 or 9 or 10 or 11 or 12 or 13 or 14 or 15 or 16 or 17 or 18 or 19 or 20 or 21 or 22 or 23 or 24 or 25 or 26 or 27 or 28 or 29 or 30 or 31 or 32 or 33 or 34 or 35 or 36 or 37 or 38 or 39 or 40 or 41 or 42 or 43 or 44 or 45 or 46 or 47 or 48 or 49 or 50 or 51 or 52 or 53 or 54 or 55 or 56 or 57 or 58 or 59 or 60 or 61 or 62 or 63 or 64 or 65 or 66 or 67 or 68 or 69 or 70 or 71 or 72 or 73 or 74 or 75 or 76 or 77 or 78 or 79 or 80 or 81 or 82 or 83 or 84 or 85 or 86 or 87 or 88 or 89

91 Treatment Guidelines/

92 Guideline*.ti.

93 Guidance.ti.

94 guidance.id.

95 (clinical adj1 guideline*).mp. [mp=title, abstract, heading word, table of contents, key concepts, original title, tests & measures]

96 (practice adj1 guideline*).mp. [mp=title, abstract, heading word, table of contents, key concepts, original title, tests & measures]

97 91 or 92 or 93 or 94 or 95 or 96

98 90 and 97

## 5 SCOPUS Search Strategy

( ( TITLE-ABS-KEY ( coproduction OR co-production ) ) OR ( TITLE-ABS-KEY ( ( stakeholder* OR advisor* OR reference* OR expert* OR consultation* OR steering ) W/2 ( group* OR panel* ) ) ) OR ( TITLE-ABS-KEY ( ( stakeholder* OR patient* OR consumer* OR public OR caregiver* OR care-giver* OR communit* OR citizen* OR user* OR service-user* OR end-user* OR clinician* OR doctor* OR physician* OR nurse* OR policymaker* OR policy-maker* OR funder* OR industr* OR pharmaceutical ) W/2 ( engag* OR involv* OR input OR participat* OR collaborat* ) ) ) ) AND ( ( TITLE ( guideline* ) ) OR ( TITLE ( guidance ) ) OR ( KEY ( guidance ) ) OR ( TITLE-ABS-KEY ( "clinical guideline*" OR "clinical practice guideline*" ) ) )

## 6 Sociological Abstracts Search Strategy

Searched for: (((MAINSUBJECT.EXACT ("Audience participation") OR MAINSUBJECT.EXACT("Participation") OR MAINSUBJECT.EXACT("Stakeholders") OR MAINSUBJECT.EXACT("Community") OR MAINSUBJECT.EXACT("Citizen participation") OR MAINSUBJECT.EXACT("Consumers")) OR noft((stakeholder* OR advisor* OR reference* OR expert* OR consultation* OR steering) NEAR/2 (group* OR panel*)) OR noft((stakeholder* OR patient* OR consumer* OR public OR caregiver* OR care-giver* OR communit* OR citizen* OR user* OR service-user* OR end-user* OR clinician* OR doctor* OR physician* OR nurse* OR policymaker* OR policy-maker* OR funder* OR indust* OR pharmaceutical) NEAR/2 (engag* OR involv* OR input OR participat* OR collaborat*))) AND (MAINSUBJECT.EXACT("Guidelines") OR ti(Guideline*) OR noft(Guidance*) OR noft(Clinical NEAR/3 guideline*))) AND stype.exact("Scholarly Journals")
